# Development of Hydrophilic Interaction Liquid Chromatography Method for the Analysis of Moxonidine and Its Impurities

**DOI:** 10.1155/2016/3715972

**Published:** 2016-10-26

**Authors:** Slavica Filipic, Milica Elek, Marija Popović, Katarina Nikolic, Danica Agbaba

**Affiliations:** Department of Pharmaceutical Chemistry, University of Belgrade, Vojvode Stepe 450, Belgrade, Serbia

## Abstract

Fast and simple hydrophilic interaction liquid chromatography (HILIC) method was developed and validated for the analysis of moxonidine and its four impurities (A, B, C, and D) in pharmaceutical dosage form. All experiments were performed on the* Agilent Technologies 1200 *high-performance liquid chromatography (HPLC) system using Zorbax RX-SIL, 250 mm × 4.6 mm, 5 *μ*m column as stationary phase (*T* = 25°C, *F* = 1 mL/min, and *λ* = 255 nm), and mixture of acetonitrile and 40 mM ammonium formate buffer (pH 2.8) 80 : 20 (v/v) as mobile phase. Under the optimal chromatographic conditions, selected by central composite design, separation and analysis of moxonidine and its four impurities are enabled within 12 minutes. Validation of the method was conducted in accordance with ICH guidelines. Based on the obtained results selectivity, linearity (*r* ≥ 0.9976), accuracy (*recovery*: 93.66%–114.08%), precision (RSD: 0.56%–2.55%), and robustness of the method were confirmed. The obtained values of the limit of detection and quantification revealed that the method can be used for determination of impurities levels below 0.1%. Validated method was applied for determination of moxonidine and its impurities in commercially available tablet formulation. Obtained results confirmed that validated method is fast, simple, and reliable for analysis of moxonidine and its impurities in tablets.

## 1. Introduction

Moxonidine belongs to the second generation of centrally acting antihypertensive drugs that exhibit high binding affinity for I_1_-imidazoline receptor and minor activity at *α*_2_-adrenoceptors which explains the absence of adverse effects characteristic for the first generation of antihypertensives such as sedation and dry mouth. It is used in therapy as antihypertensive as well as to improve metabolic profile of patients with hypertension and diabetes type 2 or with an impaired glucose tolerance [1].

**Figure 1:**
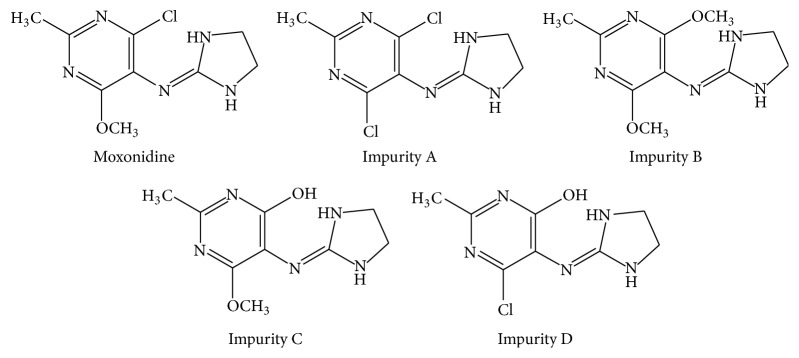
Chemical structures of moxonidine and its impurities.

The European Pharmacopoeia [2] and the British Pharmacopoeia [3] list four related substances of moxonidine: impurity A (4-chloro-moxonidine), impurity B (6-methoxy-moxonidine), impurity C (4-hydroxy-moxonidine), and impurity D (4-hydroxy-6-chloro-moxonidine). Structures of moxonidine and its impurities are shown in Figure 1. Official method in Pharmacopoeia for the determination of moxonidine and its four impurities is high-performance liquid chromatography (HPLC) based on octylsilyl silica particles as a stationary phase and the ion-pair reagents as a component of the mobile phase. From the chemical point of view moxonidine and its impurities are weak bases with nitrogen ionizable center which can be protonated. These molecules are completely ionized in an acidic medium [4] and do not have a high affinity for apolar stationary phase. In order to avoid the application of ion-pair reagent which enables adequate retention of compounds or micellar liquid chromatography (MLC) which is suitable for simultaneous determination of ionic and nonionic compounds [5, 6], analysis of ionized molecules can be performed by hydrophilic interaction liquid chromatography (HILIC) method. This method is described as a useful alternative to reverse phase chromatography in which the polar columns, such as silica and amino, are used as the stationary phase and water/buffer solution with high volume ratio of an organic solvent as a mobile phase [7, 8].

Literature survey has revealed RP-HPLC method for the determination of moxonidine in the presence of its four impurities [9] while UPLC method based on nonpolar stationary phase was used for separation and determination of moxonidine, as well as for its degradation products, which occur after the acidic, basic, or neutral hydrolysis, thermal or photolytic degradation [10]. Moxonidine was separated from its two impurities by thin layer chromatography (TLC) method [11], and fast and simple TLC method for the determination of moxonidine and its four impurities was also conducted [12].

Apart from the reported HILIC method which was applied for the separation of moxonidine and its five impurities using an amino column as the stationary phase [7], there are no HILIC reports available on the optimization of HILIC condition and determination of moxonidine and its four impurities using polar silica column. Thus, the main objective of this study was to systematically examine the retention behavior of moxonidine and its four impurities in HILIC system by central composite design and then under the optimized chromatographic conditions to validate the method for the determination of moxonidine and its four impurities in pharmaceutical dosage form according to International Council on Harmonisation (ICH) guidelines [13].

## 2. Materials and Methods

### 2.1. Reagents and Materials

Moxonidine (99.4% purity), 4-chloro-*N*-(imidazolidin-2-ylidene)-6-methoxy-2-methylpyrimidin-5-amine; impurity A (99.4% purity), 4,6-dichloro-*N*-(imidazolidin-2-ylidene)-2-methylpyrimidin-5-amine (6-chloro-moxonidine); impurity B (98.5% purity),* N*-(imidazolidin-2-ylidene)-4,6-dimethoxy-2-methylpyrimidin-5-amine (4-methoxy-moxonidine); impurity C (98.9% purity), 5-[(imidazolidin-2-ylidene)amino]-6-methoxy-2-methylpyrimidin-4-ol (4-hydroxy-moxonidine,); and impurity D (97.79% purity), 6-chloro-5-[(imidazolidin-2-ylidene)amino]-2-methylpyrimidin-4-ol (6-desmethyl-moxonidine), were obtained from Chemagis (Bnei Brak, Israel) (Figure 1). The Moxogamma® 0.4 mg film tablets were manufactured by Worwag Pharma (Böblingen, Germany).

The purified water (TKA, GenPure, Niederelbert, Germany), ammonium formate for HPLC ≥ 99.0% (Merck, Darmstadt, Germany), formic acid 98–100% for analysis (Merck, Darmstadt, Germany), and HPLC grade acetonitrile ≥ 99.93% (Sigma Aldrich, Steinheim, Germany) were used for the preparation of the mobile phase.

### 2.2. Chromatographic Conditions

Chromatographic analysis was performed using an Agilent Technologies 1200 HPLC system (Santa Clara, CA, USA) consisting of a binary pump, degasser, a thermostat for the column, and the photodiode array detector. Samples were injected through a Rheodyne injector valve with a 20 *μ*L sample loop. The analytical column Zorbax RX-SIL, 250 mm × 4.6 mm, 5 *μ*m (Agilent Technology, Santa Clara, CA, USA) was used as the stationary phase. The mobile phase consisted of a mixture of acetonitrile and 40 mM ammonium formate buffer pH = 2.8 (80 : 20 v/v). The column temperature (*T* = 25°C), the flow rate of the mobile phase (*F* = 1 mL/min), and the wavelength (*λ* = 255 nm) were kept constant during the analysis.

### 2.3. Experimental Design

The experimental scheme was obtained by central composite rotable design using Design-Expert 7.0.0 program (Stat-Ease, Minneapolis, MN, USA). Based on the observation obtained during the preliminary studies, three factors, that is, percent of acetonitrile in mobile phase (*x*_1_) examined at levels 70%, 75%, and 80%; pH of the aqueous phase (*x*_2_) examined at levels 2.8, 3.5 and 4.2; and concentration of ammonium formate in aqueous phase (*x*_3_) examined at levels 20, 40, and 60 mM were selected for screening. The soft independent modeling of class analogy SIMCA-P+ 12.0 program [14] was used for investigation of the influence of the examined factors on the retention behavior of the tested compounds. Retention factors of moxonidine and its four impurities (*k*_M_, *k*_A_, *k*_B_, *k*_C_, *k*_D_) and resolution between impurities A and B, Rs_A/B_, as well as impurities C and D, Rs_C/D_, were used as dependent variables *Y* while examined factors and their interactions were used as independent variables *X* (*x*_1_, *x*_2_, *x*_3_, *x*_1_ × *x*_1_, *x*_2_ × *x*_2_, *x*_3_ × *x*_3_, *x*_1_ × *x*_2_, *x*_1_ × *x*_3_, *x*_2_ × *x*_3_) during PLS modeling.

### 2.4. Solutions

#### 2.4.1. Preparation of Stock Solutions and Working Standard Solutions

The stock standard solutions of moxonidine (1 mg/mL), impurities A and B (0.05 mg/mL), and impurities C and D (0.01 mg/mL) were prepared separately in mixture of methanol-water (50 : 50 v/v). Stock solutions were further diluted to obtain a mixture of 100 *μ*g/mL of moxonidine, 0.5 *μ*g/mL of impurities A and B, and 1.0 *μ*g/mL of impurities C and D.

#### 2.4.2. Preparation of Solutions for the Selectivity Estimation

Placebo consisting of magnesium stearate, lactose monohydrate, povidone K-25, and crospovidone was prepared in the concentration ratio corresponding to the content in the Moxogamma 0.4 tablets. Prepared mixture was conducted through the same procedure as the tablet mass used for preparation of sample solution.

A standard solution mixture containing 100 *μ*g/mL of moxonidine, 0.5 *μ*g/mL of impurities A and B, and 1.0 *μ*g/mL of impurities C and D was used for estimation of the method selectivity.

#### 2.4.3. Preparation of Solutions for the Linearity Estimation

For the calibration curves, stock solutions were diluted with mobile phase in order to obtain nine solutions of impurities in the concentration ranges of 0.04–0.6 *μ*g/mL for impurities A and B, 0.08–1.2 *μ*g/mL for impurities C and D, and six solutions containing moxonidine in the range 25–150 *μ*g/mL.

#### 2.4.4. Preparation of the Solutions for the Accuracy and Precision Estimation

Solutions for the method accuracy and precision estimation were prepared by spiking placebo with moxonidine at concentration levels 80%, 100%, and 120% as well as with moxonidine and impurities A, B, C, and D at concentration levels corresponding to LOQ, 100% and 120%. Solutions were prepared by mixing 0.25 g of placebo with appropriate volumes of stock solutions in 10 mL volumetric flasks. After addition of 5 mL of mobile phase, solutions were treated on ultrasonic bath for 10 minutes and diluted with the mobile phase to volume. The resulting solutions were centrifuged at 3000 rpm for 15 min and then the supernatant was separated and filtered through a membrane filter (0.45 *μ*m). For each concentration level three solutions were prepared.

#### 2.4.5. Sample Preparation

Twenty tablets from which the film had previously been removed were weighted and pulverized. The amount of the tablet mass containing 1 mg of moxonidine was dissolved in 5 mL of mobile phase in 10 mL volumetric flask, sonicated for 10 min, diluted with the mobile phase to the volume, and centrifuged at 3000 rpm for 15 min. The obtained supernatant was separated and filtered through a membrane filter (0.45 *μ*m).

## 3. Results and Discussion

### 3.1. Optimization of the Mobile Phase

Moxonidine and its impurities contain guanidine function in which nitrogen atom can be protonated; thus these compounds behave as weak bases with calculated *pK*_*a*_ values 7.92, 7.06, 7.48, 7.17, and 6.95 for moxonidine, impurity A, impurity B, impurity C, and impurity D, respectively [4]. Under the acidic experimental conditions (pH 2.8) all the studied compounds are completely ionized and exist in cationic forms with the charge +1. In addition, these compounds exhibit similar polarity, with calculated log *p* values 1.77, 2.49, 1.60, 1.57, and 2.01 for moxonidine, impurity A, impurity B, impurity C, and impurity D, respectively [4]. Impurity A has the most pronounced lipophilic properties due to the presence of 2 chlorine atoms. According to increasing lipophilicity moxonidine and its impurities can be represented in the following order: C < B < moxonidine < D < A. Due to the similarity of the analyzed compounds in structure and polarity the separation of such compounds in a short period of time is a challenge for the analysts. Thus, finding appropriate separation conditions requires careful selection of a stationary and mobile phase. For optimization of chromatographic condition hydrophilic interaction liquid chromatography method using polar silica column was applied.

Preliminary experiments showed that components of the mobile phase such as content of acetonitrile, pH, and the concentration of the buffer are the factors influencing resolution and retention behavior of examined compounds. Content of acetonitrile in the mobile phase was investigated in the range of  70% to 80%. Higher percents of acetonitrile were associated with a significant extension of the analysis time, whereas the smaller proportions were drastically reduced resolution among the tested compounds. The range of the tested pH values was set between 2.8 and 4.2. With increasing pH value there was a significant prolongation of the retention time of the observed compounds. The selected concentration range of the buffer was from 20 mM to 60 mM. For the assessment of an impact of selected factors on the retention behavior of tested compounds central composite design was selected with the total number of the experiments being 20, with six experiments representing replications in the central point. Retention factors of analyzed compounds and resolution between critical peak pairs (A/B and C/D) were followed as the systems outputs. The experimental conditions designed by the experimental plan are presented in Table 1. High values of statistical parameters such as *R* ^2^ (square of the correlation coefficient) and *Q* ^2^ (cross-validated correlation coefficient) obtained for created PLS models (Table 2) ensured their good prognostic capacity.

**Figure 2:**
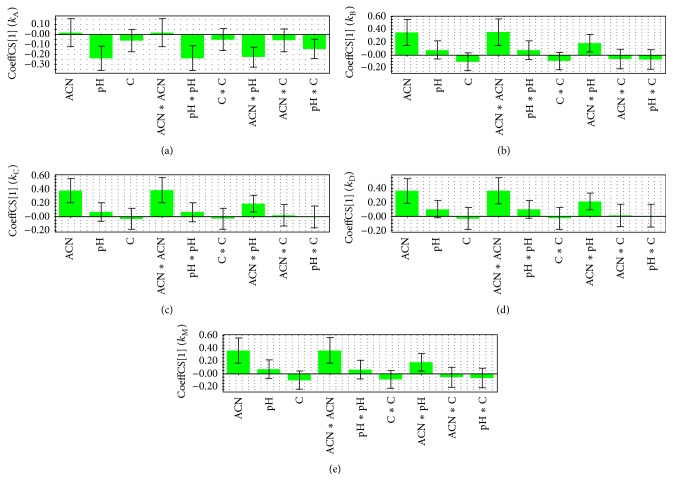
Plot of coefficients for the response variables: (a) *k*_A_, (b) *k*_B_, (c) *k*_C_, (d) *k*_D_, and (e) *k*_M_.

**Figure 3:**
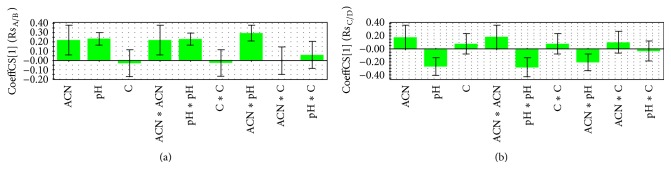
Plot of coefficients for the response variables: (a) Rs_A/B_ and (b) Rs_C/D_.

The influence of the examined factors on the retention and resolution is presented on the coefficients plots (Figures 2 and 3). On these plots, in which the regression coefficients appear as bars and the confidence intervals at 95% confidence limit as error lines, significance of different variables can be seen. The variable is considered as insignificant if the error line crosses the *x*-axis and the error is higher than the regression coefficient bar. Coefficients on the upper side of *x*-axis have a positive impact on examined output variable, while coefficients on the bottom side of *x*-axis have a negative impact on examined output variable.

The coefficient plot (Figure 2) of the PLS (*k*_A_) model indicates that all significant variables (pH, pH × pH (nonlinear effect), ACN × pH (interaction effect), pH × C (interaction effect)) are in negative correlation with retention factor of impurity A, while for all other tested compounds same significant variables (ACN, ACN × ACN (nonlinear effect), ACN × pH (interaction effect)) appeared as important factors influencing their retention and were in positive correlation with *k*_B_, *k*_C_, *k*_D_ and *k*_M_ (Figure 2).

Among individually tested components of mobile phase, acetonitrile had the highest effect on the retention behavior of moxonidine and impurities B, C, and D. Increasing the content of acetonitrile leads to an increase values of retention factors and to higher retention of substances on the column. On the other hand, change in pH value was the most important factor influencing the retention of impurity A (Figure 2).

Statistically best models obtained when the resolution between impurities A and B, Rs_A/B_, as well as impurities C and D, Rs_C/D_, was followed as the responses were the PLS models with the following statistical parameters: PLS (Rs_A/B_): *R* ^2^ = 0.897 and *Q* ^2^ = 0.833; PLS (Rs_C/D_): *R* ^2^ = 0.802 and *Q* ^2^ = 0.71.

Upon examination of the coefficient plot of the PLS models (Figure 3), the most important influences on the resolution between critical peak pairs (impurities A and B) were shown: ACN, pH, ACN × ACN (nonlinear effect), ACN × pH (effect of interaction), and pH × pH (nonlinear effect). All of these variables are in positive correlation with Rs_A/B_. The highest influence on resolution between peaks C and D was shown: pH, pH × pH (nonlinear effect), and ACN × pH (effect of interaction) and all were in negative correlation with (Rs_C/D_). Performed experiments revealed that combination of lower percent of acetonitrile (70%) and lower pH values (2.8) regardless of buffer concentration leads to coelution of impurities A and B (Rs_A/B_ = 0), while critical separation of impurities C and D (Rs_C/D_ = 0) has been observed under pH values 4.68, 75% of acetonitrile, and 40 mM buffer. These critical experimental conditions for impurities C and D were suitable for separation of impurities A and B whose resolution was 10.91. Under the all examined conditions given on Table 1 resolution between impurities A and B was significantly more sensitive to the changes of tested factors compared to resolution between impurities C and D. Finally, for the most examined experimental conditions both critical resolutions were higher than 2, but detailed analysis of the influence of various factors on the retention behavior of the tested compounds revealed that the optimal chromatographic conditions for the separation of moxonidine and its four impurities can be achieved using a mobile phase consisted of acetonitrile, 40 mM buffer solution, pH 2.8 (80 : 20 v/v) at a temperature of 25°C, flow rate of 1 mL/min, and at a wavelength of 255 nm. The optimized method was further validated in order to confirm its selectivity, linearity, precision, accuracy, sensitivity, and robustness, as well as the possibility for applying in determination of moxonidine and its impurities in pharmaceutical dosage form.

### 3.2. Method Validation

#### 3.2.1. Selectivity

**Figure 4:**
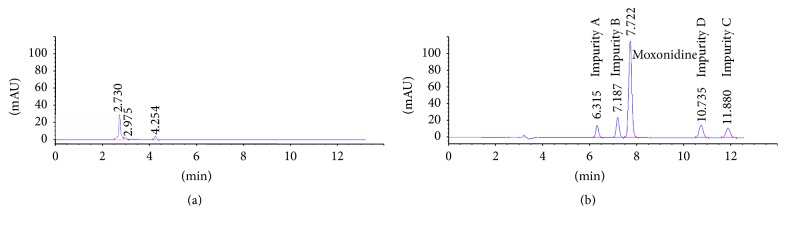
The chromatograms showing the estimation of method selectivity: (a) a chromatogram of placebo solution; (b) a chromatogram of standard mixture.

The selectivity of the method was proved by comparing the chromatograms of placebo mixture and standard solution mixture. At the retention times of the analytes no significant interfering peaks originating from the placebo sample were noted (Figure 4).

#### 3.2.2. The Limit of Detection (LOD) and Limit of Quantification (LOQ)

Experimentally determined values of limit of detection (LOD) and limit of quantification (LOQ) for impurities A, B, C, and D were defined according to signal-to-noise ratio (S/N) corresponding to 3 : 1 for LOD and 10 : 1 for LOQ. The obtained values of LOD were 0.012 *μ*g/mL for impurities A and B (corresponding to 0.012%) and 0.024 *μ*g/mL for impurities C and D (corresponding to 0.024%) and of LOQ 0.04 *μ*g/mL for impurities A and B (corresponding to 0.04%) and 0.08 *μ*g/mL for impurities C and D (corresponding to 0.08%).

#### 3.2.3. Linearity

The calibration curves of peak areas against concentrations were linear in the investigated range (from LOQ to 120% of intended test concentration for impurities A, B, C, and D and from 25–150% for moxonidine) with correlation coefficients higher than 0.997 (Table 3). Besides, statistical significance of the intercepts (*t*) was not higher than tabular values (*t*_0.05_) which confirmed the absence of interferences (Table 3).

#### 3.2.4. Precision and Accuracy

The assessment of method precision was done by calculating the relative standard deviation (RSD) for each target concentration level. The obtained values are presented in Table 4 and fulfilled the required criteria (RSD 2% for the active substance, 10% for impurities C and D, and 15% for impurities A and B) [15].

The accuracy of the method was evaluated according to the obtained recovery values (Table 4). For all tested concentration levels obtained recoveries for impurities were in the range 93.66%–114.08% and for moxonidine 99.04%–101.54% which meets the requirements for the method accuracy (98%–102% for active ingredients, 70.0–130.0% for impurities 0.1% < *x* < 0.5%, or 80.0–120.0% for impurities 0.5% < *x* < 1.0%) [15].

#### 3.2.5. Robustness

Robustness is a measure of the capacity of the method to remain unaffected by small yet deliberate variations of working conditions, and it is indicative of the method reliability. ICH Q2 (R1) guideline provides some recommendations for the factors that should be examined during robustness testing [13]. In this study, robustness of the method was estimated by applying small variations of chromatographic conditions such as column temperature 25 ± 2°C, flow rate 1.0 ± 0.1 mL/min, buffer pH 2.8 ± 0.05, and the volume ratio of acetonitrile in the mobile phase ±0.5%. During examination of the robustness one-factor-at-a-time approach was applied, which means that one factor is changed while others were kept on constant level. The highest impact on chromatographic behavior of tested compounds showed % of acetonitrile in mobile phase. Finally, all defined variations in comparison with optimal chromatographic condition did not affect significantly changes in peak areas (less than 5%), retention times (less than 3%), and resolution (less than 3%) between the tested compounds indicating that method is robust.

#### 3.2.6. Application of the Method in the Moxogamma 0.4 Tablet Analysis

In order to confirm the applicability of validated method, the proposed method was applied in the analysis of commercially available Moxogamma 0.4 tablet. The obtained results (97.5% for content of moxonidine, 0.68% for impurity C, 0.87% for impurity D, and below LOQ values for impurities A and B) were in accordance with manufacture specification (impurities A and B below 0.5% and impurities C and D below 1%).

### 3.3. Advantages of the Method

As noted above, by applying the HILIC method in which polar silica column was used as stationary phase complete separation of moxonidine and its four impurities has been achieved for only 12 minutes. All tested compounds which are positively charged under the examined chromatographic conditions accomplished adequate retention which is often difficult to achieve without the use of ion-pair reagent. In this way the extension of chromatographic analysis has been avoided, not only in terms of duration of chromatographic run but also in terms of column conditioning and washing. Selected temperature and mobile phase composition are not aggressive and have favorable influence on the column lifetime. In addition, validated method is sensitive and enables determination of impurities present at 0.04% (impurities A and B) and 0.08% (impurities C and D) level.

## 4. Conclusion

Retention behavior of moxonidine and its four impurities has been examined by using a central composite design. The most important factors and their interactions with a highest influence on resolution between critical peaks pairs were determined and optimal chromatographic conditions for separation of moxonidine and its four impurities were achieved. The proposed method is selective, linear, accurate, precise, and robust and has been applied for the determination of moxonidine and its impurities in the commercially available pharmaceutical dosage form. The obtained results showed that content of moxonidine and its four impurities meet the requirements of manufacturers.

## Figures and Tables

**Table 1:** Plan of experiments.

Exp. number	% ACN	pH of the buffer solution	Concentration of ammonium formate (mM)
1	75	3.5	40
2	75	3.5	40
3	75	3.5	40
4	75	3.5	40
5	75	3.5	40
6	75	3.5	40
7	70	2.8	20
8	80	2.8	20
9	70	4.2	20
10	80	4.2	20
11	70	2.8	60
12	80	2.8	60
13	70	4.2	60
14	80	4.2	60
15	66.59	3.5	40
16	83.41	3.5	40
17	75	2.32	40
18	75	4.68	40
19	75	3.5	6.36
20	75	3.5	73.64

**Table 2:** Statistical parameters of created PLS models.

PLS models	*R* ^2^	*Q* ^2^
PLS (*k*_A_)	0.648	0.584
PLS (*k*_B_)	0.792	0.707
PLS (*k*_C_)	0.983	0.964
PLS (*k*_D_)	0.98	0.959
PLS (*k*_M_)	0.81	0.731
PLS (Rs_A/B_)	0.897	0.833
PLS (Rs_C/D_)	0.802	0.71

**Table 3:** Statistical data for the calibration curves.

Compound	Concentration range (*μ*g/mL)	Regression equations	*r*	*t*	*t* tab (*p* = 0.05)
Moxonidine	25–150	*y* = 29142.47*x* + 94.864	0.9992	1.1968	2.365
Impurity A	0.04–0.6	*y* = 24.45681*x* − 0.09526	0.9991	0.0607	2.262
Impurity B	0.04–0.6	*y* = 27.659*x* + 0.1724	0.9976	0.5319	2.262
Impurity C	0.08–1.2	*y* = 18.18141*x* + 0.1283	0.9982	0.4016	2.262
Impurity D	0.08–1.2	*y* = 11.82117*x* + 0.1404	0.9976	0.5762	2.262

**Table 4:** Accuracy and precision of the method.

Compound	Concentration level (%)	Concentration (*μ*g/mL)	Recovery (%)	RSD (%)
Moxonidine	80	80	99.04	1.85
	100	100	101.15	0.45
	120	120	101.54	0.32

Impurity A	LOQ	0.04	95.89	3.79
	100	0.5	101.22	0.35
	120	0.6	100.51	0.88

Impurity B	LOQ	0.04	97.65	2.92
	100	0.5	101.28	0.27
	120	0.6	99.19	0.85

Impurity C	LOQ	0.08	95.95	2.02
	100	1.0	114.08	0.27
	120	1.2	93.82	1.48

Impurity D	LOQ	0.08	93.66	2.09
	100	1.0	100.83	0.83
	120	1.2	97.77	0.39

## References

[1] Fenton C., Keating G. M., Lyseng-Williamson K. A. (2006). Moxonidine, a review of its use in essential hypertension. *Drugs*.

[2] (2014). *European Pharmacopoeia*.

[3] British Pharmacopoeia (2011). *British Pharmacopoeia Commission*.

[4] ChemAxon (2013). *MarvinSketch 6.1.0*.

[5] Esteve-Romero J., Carda-Broch S., Gil-Agustí M., Capella-Peiró M.-E., Bose D. (2005). Micellar liquid chromatography for the determination of drug materials in pharmaceutical preparations and biological samples. *TrAC—Trends in Analytical Chemistry*.

[6] Carda-Broch S., Esteve-Romero J., García-Alvarez-Coque M. C. (2000). Furosemide assay in pharmaceuticals by micellar liquid chromatography: study of the stability of the drug. *Journal of Pharmaceutical and Biomedical Analysis*.

[7] Olsen B. A. (2001). Hydrophilic interaction chromatography using amino and silica columns for the determination of polar pharmaceuticals and impurities. *Journal of Chromatography A*.

[8] Buszewski B., Noga S. (2012). Hydrophilic interaction liquid chromatography (HILIC)-a powerful separation technique. *Analytical and Bioanalytical Chemistry*.

[9] Milovanović S., Otašević B., Zečević M., Živanović L., Protić A. (2012). Development and validation of reversed phase high performance liquid chromatographic method for determination of moxonidine in the presence of its impurities. *Journal of Pharmaceutical and Biomedical Analysis*.

[10] Otašević B., Milovanović S., Zečević M., Golubović J., Protić A. (2014). UPLC method for determination of moxonidine and its degradation products in active pharmaceutical ingredient and pharmaceutical dosage form. *Chromatographia*.

[11] Kakde R., Gadpayale K., Qureshi M. O. (2012). Stability indicating HPTLC method for determination of Moxonidine in pharmaceutical preparations. *International Journal of PharmTech Research*.

[12] Filipic S., Shenger M. S. M., Nikolic K., Agbaba D. (2015). Determination of moxonidine and its impurities by thin-layer chromatography. *Journal of Liquid Chromatography & Related Technologies*.

[13] Guideline, ICH Harmonized Tripartite, “Validation of analytical procedures: text and methodology”*, Q2 (R1) 1*, 2005

[14] Umetrics AB http://umetrics.com/.

[15] Crowther J. B., Ahuja S., Scypinski S. (2001). Validation of pharmaceutical test methods. *Handbook of Modern Pharmaceutical Analysis*.

